# Mechanical Lock Joint for Effective In-Plane Application of Concentrated Loads to Thin Polymer Matrix Laminates

**DOI:** 10.3390/polym13111762

**Published:** 2021-05-27

**Authors:** Piotr Czarnocki, Tomasz Zagrajek, Jan Tomasiewicz

**Affiliations:** Institute of Aeronautics and Applied Mechanics, Warsaw University of Technology, Nowowiejska 24, 00-665 Warsaw, Poland; tzagra@meil.pw.edu.pl (T.Z.); jan.tomasiewicz@meil.pw.edu.pl (J.T.)

**Keywords:** metal-composite joint, strength, load transfer mechanism

## Abstract

Means of in-plane loading of thin laminates with concentrated loads are of high practical importance. The purpose of this work was to investigate experimentally and numerically the mechanism of load transfer, load capacity, damage and associated failure modes of a specific, mechanical lock joint intended for in-plane loading of thin laminate plates with concentrated loads. The experimental investigations were carried out with the digital image corelation (DIC) and computed tomography (CT), and numerical ones with the help of a non-linear FE modelling, accounting for progressive damage. For this purpose, a special algorithm was developed accounting for a continuous degradation of the stiffness moduli of the laminate with strains according to the custom defined degradation law. Due to the specific design, the joint loaded a laminate plate with its front and rear parts, unlike a typical bolt joint transferring a load only by contact pressure developed at the front side of a bolt. Due to this feature, the load capacity of the joint was almost two times higher than that of a typical bolt joint of the same relevant dimensions.

## 1. Introduction

**Figure 1:**
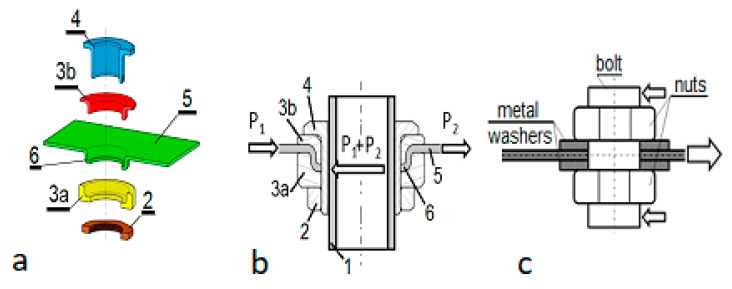
Design of the ML joint: (**a**,**b**) (1) bolt, (2) nut, (3a, 3b) collar inserts, (4) flanged sleeve with threaded end, 5. laminate plate, (6) plate flange; (**c**) Schematic view of a simple bolt joint with washers (SBW).

Polymer matrix laminates have been widely used by a broad spectrum of industries including space and aircraft industry, e.g., [1,2], shipbuilding industry [3,4], automotive [5], railroad [6] and construction [7,8]. It is well known that the Achilles’ heel of such composites is their relatively low bearing strength, and for this reason, introduction of point loads is not an easy task. The presented work focused on a particular mechanism of load transfer, load capacity, damage and associated failure modes of a specific, mechanical lock joint intended for in-plane loading of thin laminate plates with concentrated loads and not for connecting laminate plates. The considered joint is referred to as the metal lock (ML) joint. The joint, (Figure 1a), consisted of two collar inserts (3a and 3b), a one-end thread flanged sleeve (4) and a nut (2) clamping the two collar inserts. Load P = P_1_ + P_2_ was transferred from the sleeve (4) to the laminate plate (5) with collar inserts (3a) and (3b), clamping the laminate flange (6), (Figure 1b).

Means of in-plane loading of thin laminates with concentrated loads are of high practical importance. Unfortunately, the design of an efficient joint suitable for this purpose is not an easy task. 

**Figure 2:**
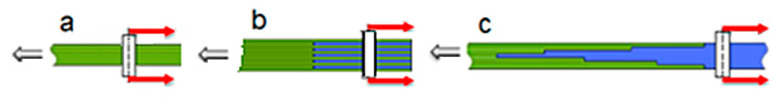
Schematic view of various designs that can be used for in-plane loading of thin laminates with the use of pins: (**a**) simple application of pin, (**b**) application of metal inserts to increase bearing strength—so called laminate hybridization [9], (**c**) use of a metal tang [16].

Very often, for this purpose mechanical joints taking advantage of pins or bolts are used (Figure 2). This would be a straightforward solution to the problem, however pins and bolts cause high bearing stress in the adjusted composite structure. Unfortunately, polymer matrix composites have relatively low bearing strength [9] (compared to metals), which makes this solution even worse. Bearing strength can be improved when bolts with washers are used (Figure 1c), thereby to a certain extent preventing fiber micro-buckling in close proximity of the bolt surface [10]. A similar effect can be achieved with the use of flanged sleeves [11]. Bearing strength of a bolt joint can also be increased by formation of a thin resin interleave with the help of the resin infusion technique [12]. Hybridization of the laminate structure surrounding a bolt can be another solution to the problem resulting from the low bearing strength of polymer matrix composites. Hybridization can be achieved with the use of thin metal sheet inserts glued between the reinforcement layers constituting the structure to be loaded [11,13,14], (Figure 2b). In the case of laminates containing reinforcement layers of different orientations, the in-plane geometry of such inserts can be adjusted to the reinforcement direction to improve load transfer [15]. Due to high stiffness and bearing strength of such inserts they can take over the load from the bolt and transfer it to each laminate ply due to the shear stress arising at the insert–laminate bond line. Additionally, specially shaped metal parts in the form of flat stepped tangs inserted at the ends of laminate plates can be used [16], (Figure 2c). Unfortunately, such designs entail manufacturing and quality control inconveniences typical for adhesive bonding. These are: (i) need for surface preparation and (ii) concern about the presence of kissing bonds being difficult for detection. It is likely that these problems could be alleviated to a certain extent through the use of metal inserts with protruding micro pins. Such designs are presented in [17] and further investigated in [18,19]. However, this technology is still in its early development stages. 

The ML joint is an alternative to the aforementioned ones. Unlike in the case of conventional designs involving pins and bolts which load a laminate with their front surfaces, the ML joint was designed to transfer the load with front and rear joint sides (forces P_1_ and P_2_, respectively, see Figure 1b). In addition, this joint does not have a majority of the above-mentioned shortcomings of conventional pin or bolt joints. In particular, it does not produce high bearing stress as opposed to the previously mentioned ones and is solely mechanical, therefore does not involve any gluing which is necessary in the case of laminate hybridization. In consequence, the ML joints do not cause problems related to the gluing, such as surface preparation and they eliminate the risk of occurrence of kissing bonds. Furthermore, the ML joint can be disassembled, which allows periodic service inspections. 

**Figure 3:**
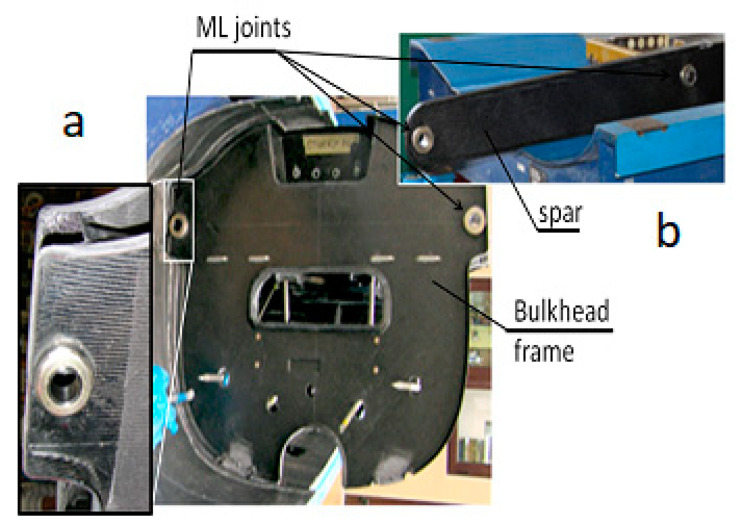
Application of the ML joint for the wing-fuselage main attachment (**a**) bulkhead, (**b**) spar.

Some examples of the ML joint applications are shown in Figure 3. Additional information can be found in, e.g., [20].

Below, experimental and numerical investigations of the ML joint are presented. The investigations were aimed at load capacity of the joint, the load transfer mechanism specific for this joint, (the way the surrounding laminate was loaded by the joint) and the related failure mode. The experimental investigations were carried out with the digital image correlation (DIC) technique [21] and with the computed tomography method (CT) [22], since both the methods are well recognized tools for deformation and failure investigations of composite structures, e.g., [23,24,25,26,27]. To obtain supplemental information concerning the load transfer mechanism specific for this joint and information about the development of the laminate damage in the vicinity of the joint, both of which were difficult to extract from the experimental work alone, the numerical investigations were carried out with the use of the FEM.

To put the performance of the ML joint in perspective, the corresponding performance of a simple bolt joint with washers (SBW), (Figure 1c), was used as a reference point, i.e., load capacity and failure modes of both joints were compared. To facilitate such comparison the bolt diameter and external washer diameter were the same as the external diameters of the sleeve and the metal collar inserts of the ML joint.

## 2. Materials and Methods

### 2.1. Experimental Investigations

#### 2.1.1. Test Piece. Materials and Design

**Figure 4:**
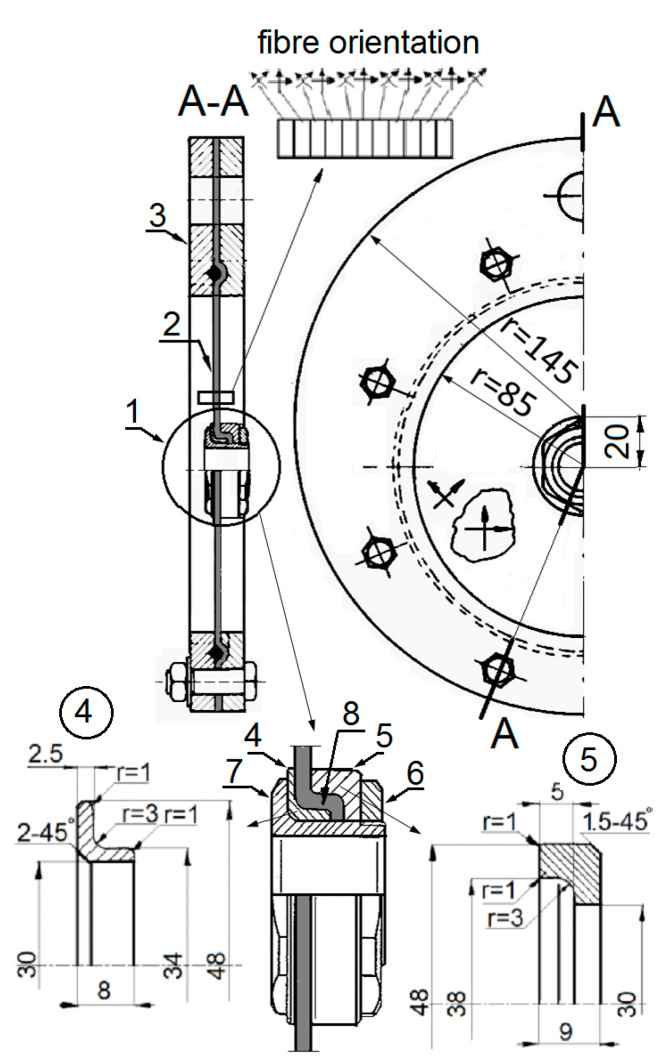
Test piece design (all dimensions in mm).

The parts of the joint were made of 30HGSA alloy steel. The laminate was made of MTM46/CF0305 CF/epoxy fabric prepreg of Solvay [28], designed for the vacuum bag only, (VBO), curing process. The prepreg was reinforced with 2 × 2 twill fabric. Its areal weight was 199 g/m^2^ and the corresponding resin content was 42% RW. The laminate consisted of 11 layers of such a prepreg, stacked according to the following pattern: 45°/0°/45°/0°/45°/0°/45°/0°/45°/0°/45° (the angles indicate orientation of the fabric warp relative to the loading direction). To form the plate flange 8, (Figure 4), a laminate plate was clamped between the collar inserts (4 and 5) before curing. This forced the clamped portion of the plate to conform to the shape of the collar inserts. Prior to this action, the inserts’ surfaces were covered with a release agent to prevent their sticking to the uncured laminate and to allow for future disassembling of the joint, when needed. The laminate was initially cured for 5 h at 60 °C and post-cured for 5 h at 130 °C.

#### 2.1.2. Experimental Setup

**Figure 5:**
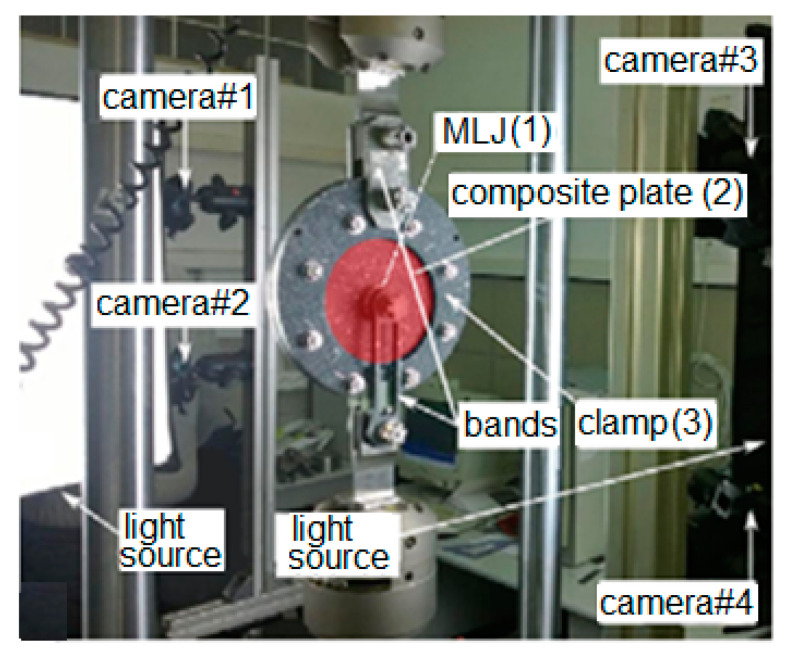
Experimental setup for DIC measurements.

The experimental setup is shown in Figure 5. It consisted of the ML joint (1), the circular layered CF/epoxy plate which was to be loaded, (2) the external metal clamps, and (3) holding the plate. The load was applied via the bolts inserted into the ML joint (1) and the external clamp (3). The bolts themselves were loaded with the use of metal bands connected to the grip of an Instron 8250 test machine. The test was run at room temperature with the crosshead speed of 0.25 mm/min.

The deformations of the plate in the x, y and z directions were experimentally determined with the DIC technique. It allowed visualization of the strain field and, on this basis, deduction of the way in which the ML joint was loading the plate, as well as construction of the load–displacement curve needed for the assessment of the load-transfer capacity of the joint. The pictures were taken with the use of four Point Gray Grasshopper cameras. Each of them was equipped with a 2448 × 2048 pixels matrix and a lens of focal length 8 mm. Such a setup allowed the authors to perform displacement measurements with 2 μm accuracy. The pictures were taken every one second. 

The specimen was loaded up to the local failure of the plate in the vicinity of the ML joint. The failure was marked by a significant drop of the specimen load capacity, clearly displayed by the load–displacement curve. 

To investigate the damage, a 50 × 50 mm piece of the plate surrounding the ML joint was cut off for the CT inspection. For the inspection a *v*|*tome*|*x* tomograph from GE was used. (Lamp: voltage 100 kV or 180 kV/15 W and current 100–180 μA, detector GE dynamic 41/200—2000 × 2000 pixel, voxel size 102–181 μm and number of projections 1440).

The SBW joint was tested in a similar manner. The only difference consisted in that the displacement of the bolt relative to the clamp was only measured with a dial gauge.

### 2.2. Numerical Modelling

A large number of numerical methods have been developed to model progressive damage of laminates loaded via a pin or bolt joint. These methods involve an application of certain failure criteria to trigger the failure process and the rules defining the failure progress. Often, for laminates made of UD prepregs, Hashin type criteria are used, e.g., [29,30,31], as well as ones such as LaRC03 and LaRc04, e.g., [32,33]. In the case of the cited works, the damage was simulated by introducing some damage parameters reducing the values of the stiffness matrix components. These parameters could assume just two constant values: 1 for a pristine material and close to 0, if the chosen failure criterion was met [29]. Additionally, some other, but still constant values of the damage parameters could be assumed [31]. Comparison of the results obtained with the maximum stress and the Hashin type criteria can be found in [34]. Additionally, for progressive damage modelling, cohesive zone elements can be used [35]. A more complex degradation process can be modelled by varying damage parameters in a linear manner according to some assumed formulas, e.g., [30,32,33].

#### 2.2.1. Finite Element Model

The presented FE model was developed with the use of ANSYS v.15 [36] and accounted for a nonlinear continuous variation of damage parameters with strains according to the original formulas (1)–(4). For this purpose, an original subroutine comprising several MACROs was developed in the APDL language. 

The MACROs carried out the following operations:

- reading the average of strain components (6 components) in all the layers (11 layers);
- comparing the readings with the failure values;
- calculating the new values of E and G according to the degradation rules, Equations (2) and (4), if the failure strain values were reached;
- replacing the old E and G values with the new ones,
- repeating the entire calculation with no external loading change, if E and/or G were changed at least in one element’s layer; and
- increasing the external load by the prescribed value and repeating the entire calculation if both the E and G values remained not changed.

To trigger the damage, the maximum strain criterion was used. The rationale for choosing this instead of Hashin’s criterion was the fact that the investigated laminate was made of symmetric fabrics. Hashin developed his criterion for transversely isotropic materials [37]. A UD laminate can be considered as such, while a laminate reinforced with symmetric fabrics cannot. Application of the Hashin type failure criteria to predict the damage onset in fabric reinforced laminates was questioned in [34,38,39]. Furthermore, it was shown that the application of much simpler maximum stress criterion (which is similar to the maximum strain criterion), yielded better results. Although, in its present version the model developed by the authors was less advanced in terms of the applied failure criterion triggering the damage process than those presented in the above-mentioned literature, nevertheless, more complex failure criteria, e.g., Hashin and similar, could be incorporated into the developed algorithm if a UD reinforcement were used.

It will be shown in Section 4 that despite the simplicity of the applied FE model, the obtained numerical results were in reasonably good agreement with the experimental ones. 

**Figure 6:**
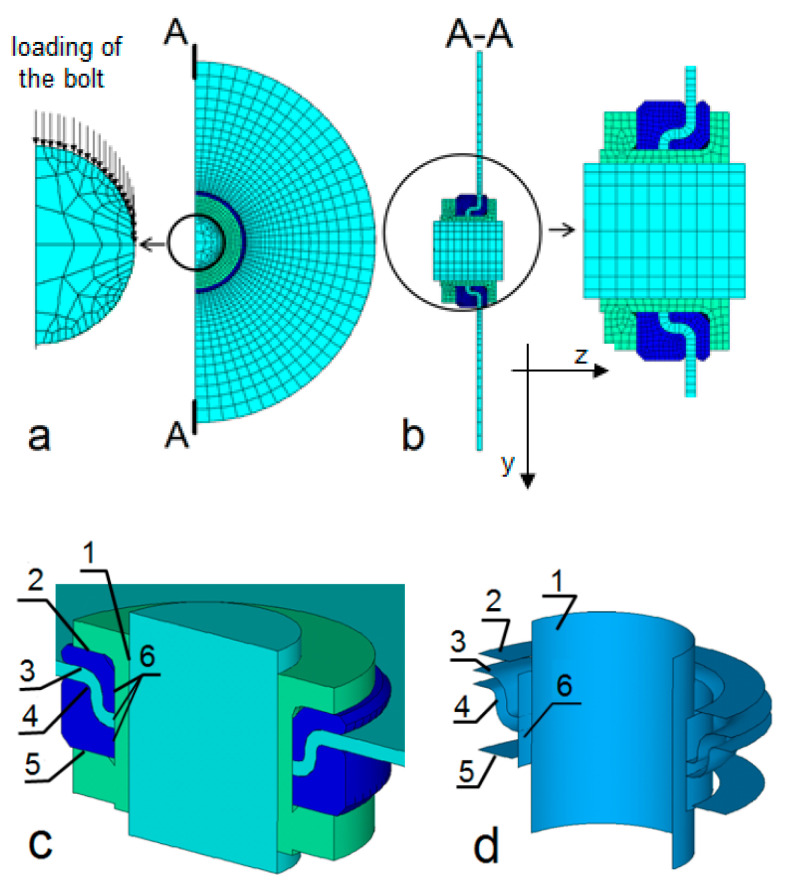
FE model of the ML joint: (**a**,**b**) mesh design; (**c**,**d**) location and visualization of the interfaces containing contact pair elements consisting of CONTA173 and TARGET170 elements; in Figure 6a, the arrows represent schematically the bolt loading due to the prescribed and the same node displacement of each node being located on the upper halves of the bolt’s external edges.

A general view of the FE model is presented in Figure 6. The external clamps marked (3) in Figure 4 were not modeled; instead, to mimic the clamp plate’s interaction, all the degrees of freedom were removed for all the nodes located at the perimeter of the plate. For all the nodes located on the symmetry plane the displacements in the z direction were set to zero. To mimic the external loading, all the nodes of the upper halves of the external edges of the bolt were subjected to the same displacements in the y direction (Figure 6a). The displacement increment Δu_y_ mimicking the loading was set to 0.05 mm. A further decrease in Δu_y_ significantly increased the computational time without any meaningful improvement in the solution convergency. The loading (displacement in y direction) was increased until an excessive distortion of one or more of elements occurred, causing a lack of the solution convergence.

The plate was modelled with one layer of SOLID185 Layered Solid elements. Each of the elements consisted of 11 layers and each of the element layers represented one of the reinforcement layers composing the plate. The metal parts were modelled with SOLID185 elements. The surface-to-surface contact pair elements consisted of CONTA173 and TARGET170 elements. They were located at the laminate–metal inserts (3, 4), bolt–sleeve (1), sleeve–insert (2) and insert–nut (5) interfaces, (Figure 6c,d), to mimic the laminate–ML joint interaction and the interactions of the ML joint metal parts, including the bolt. Unique material numbers were assigned to all the element layers of all the elements the plate consisted of (11,880 materials). Each of these materials had its initial mechanical properties assigned (Table 1) and defined in the material coordinate system.

**Figure 7:**
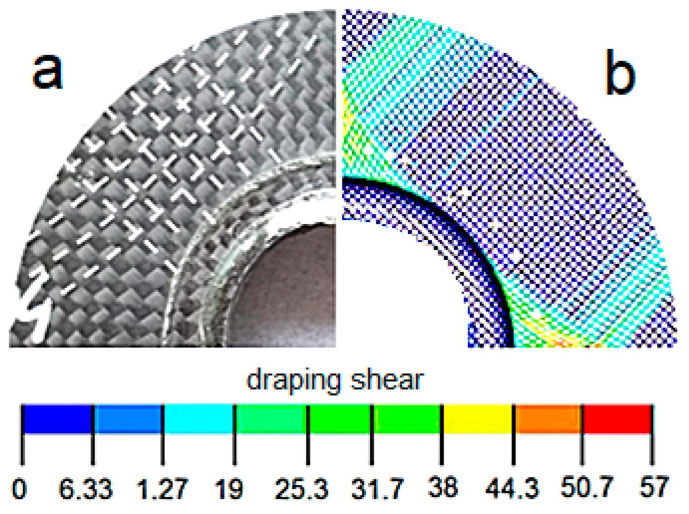
Effect of draping: (**a**) specimen, (**b**) FE model.

To approximate directional distortion of the weft and warp bundles (Figure 7), resulting from the conformity of not-yet-cured laminate to the shape of the collar inserts, a relevant modelling was carried out (draping) with the ANSYS ACP tool [36].

#### 2.2.2. Damage Simulation

**Figure 8:**
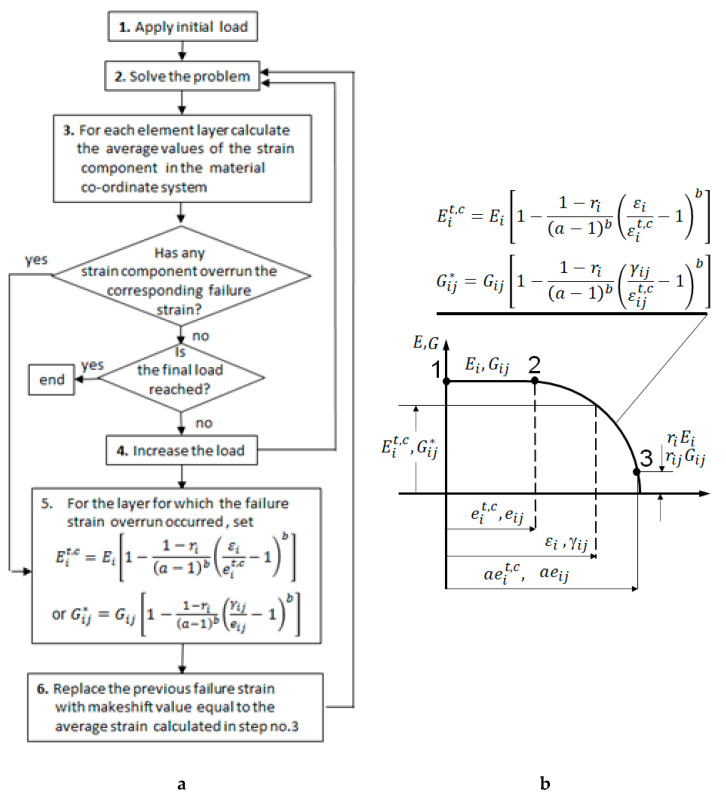
Damage simulation: (**a**) flow chart of the FE procedure, (**b**) degradation law of the elastic constants.

The development of damage was simulated with the procedure presented in the flowchart shown in Figure 8.

The maximum strain criterion was applied to each element layer of all the elements. If the criterion was met for none of the considered layers, the elastic constants remained unchanged, i.e., Equations (1) and (3) were applied, and the bolt displacement was increased by the prescribed value (step no.4). If the failure criterion was met for one or more layers composing SOLID185 element, the load applied to the bolt remained unchanged, but the elastic constant(s) corresponding to the overrun strain(s) was modified according to Equation (2) or (4), (step no.5), and then step no.2 was executed. The value of the strain component that exceeded the assumed failure strain became a makeshift failure strain for the considered element layer, (step no.6). If this strain was overrun in consecutive load steps, it would again trigger the modification of the corresponding elastics constant according to Equation (2) or Equation (4). The entire procedure was repeated until the prescribed load was reached or it was not possible to obtain a convergent solution due to extensive element distortion.

(1) $$\mathrm{if}\;\varepsilon_{i}\leq e_{i}^{t,c}\;\mathrm{then}\;E_{i}^{c}=E_{i}$$

(2) $$\mathrm{if}\;\varepsilon_{i}\geq e_{i}^{t,c}\;\mathrm{then}\;E_{i}^{t,c}=E_{i}\left[1-\frac{1-r_{i}}{{\left(a-1\right)}^{b}}{\left(\frac{\varepsilon_{i}}{e_{i}^{t,c}}-1\right)}^{b}\right]$$

(3) $$\mathrm{if}\;\gamma_{ij}\leq e_{ij}\;\mathrm{then}\;G_{ij}^{*}=G_{ij}$$

(4) $$\mathrm{if}\;\gamma_{ij}\geq e_{ij}\;\mathrm{then}\;G_{ij}^{*}=G_{ij}\left[1-\frac{1-r_{ij}}{{\left(a-1\right)}^{b}}{\left(\frac{\gamma_{ij}}{e_{ij}}-1\right)}^{b}\right]$$

where:

$e_{i}^{t,c}=X_{\epsilon t},\;Y_{\epsilon t},\;Z_{\epsilon t},X_{\epsilon c},\;Y_{\epsilon c},\;Z_{\epsilon c}$ and $e_{ij}=S_{\epsilon xy},\;S_{\epsilon yz},\;S_{\epsilon xz}\;$ are failure strains, $\varepsilon_{i}$, $\gamma_{ij}$—actual strain components as determined from the FE calculation at the start of step no.5. 

These strains became makeshift failure strains if condition (2) or (4) was met

*a*, *b*, $r_{i}$, $r_{ij}$—parameters defining degradation of stiffness (arc 2-3 in Figure 7b). For the purpose of the performed analysis their values were set to *a* = 3, *b* = 4, $r_{i}=0.0005$, $r_{ij}=0.0005$ based on the previously performed model caliberation.

The calibration involved adjustment of the a and b values in Equations (2) and (4) by trial and error method to match the experimental and numerical results regarding the load–displacement relationship. 

An advantage of the presented approach over that offered by the ANSYS consisted in that the ANSYS’ procedure reduces the stiffness matrix components by a user prescribed, constant, and unchanged values, while the presented one reduces them gradually with the increasing strains, which is more realistic.

## 3. Results

**Figure 9:**
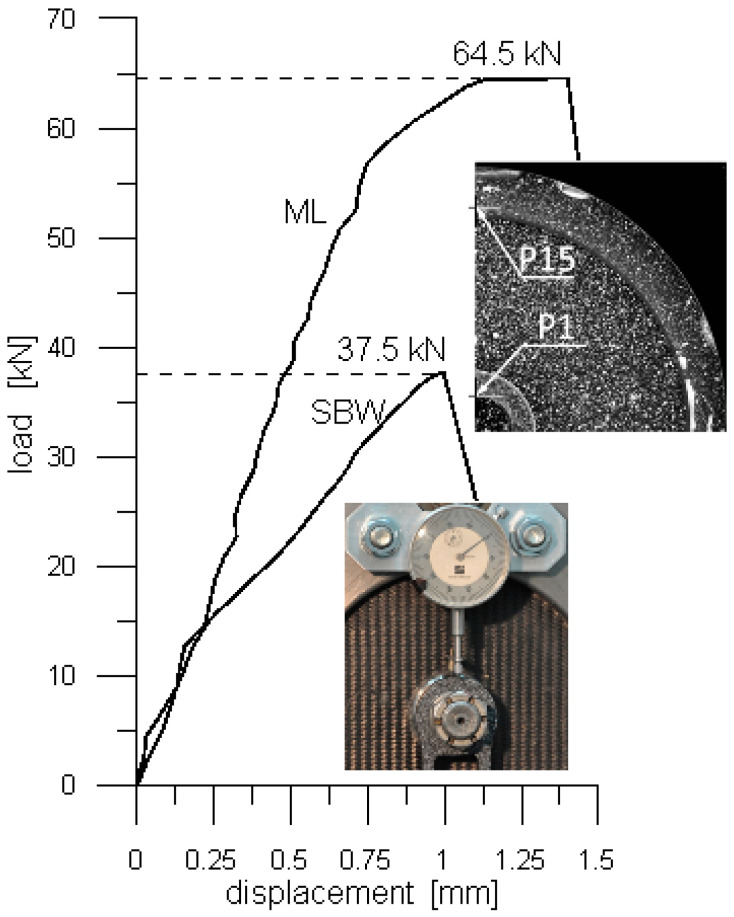
Experimentally determined load vs. displacement relationships presenting performances of the ML and SBW joints.

Experimentally determined load vs. displacement curves, presenting performances of the ML and SBW joints are shown in Figure 9. In the case of the ML joint, the DIC was used to determine relevant displacement between point P1 located at the edge of the bolt and point P15 located at the edge of the clamp (upper superimposed image, Figure 9). In the case of the SBW joint, the relative displacement of the clamp and the washer was measured with a dial gauge. The dial gauge housing was fixed to the one of clamps, and the probe tip contacted the top surface of the washer, (lower superimposed image, Figure 9). The initial stiffness of both the joints was the same, however, at about 25% of the load capacity of the ML joint, an abrupt stiffness drop in the SWB joint occurred marking initiation of damage, while the stiffens of the former remained unchanged to about 90% of its load capacity.

**Figure 10:**
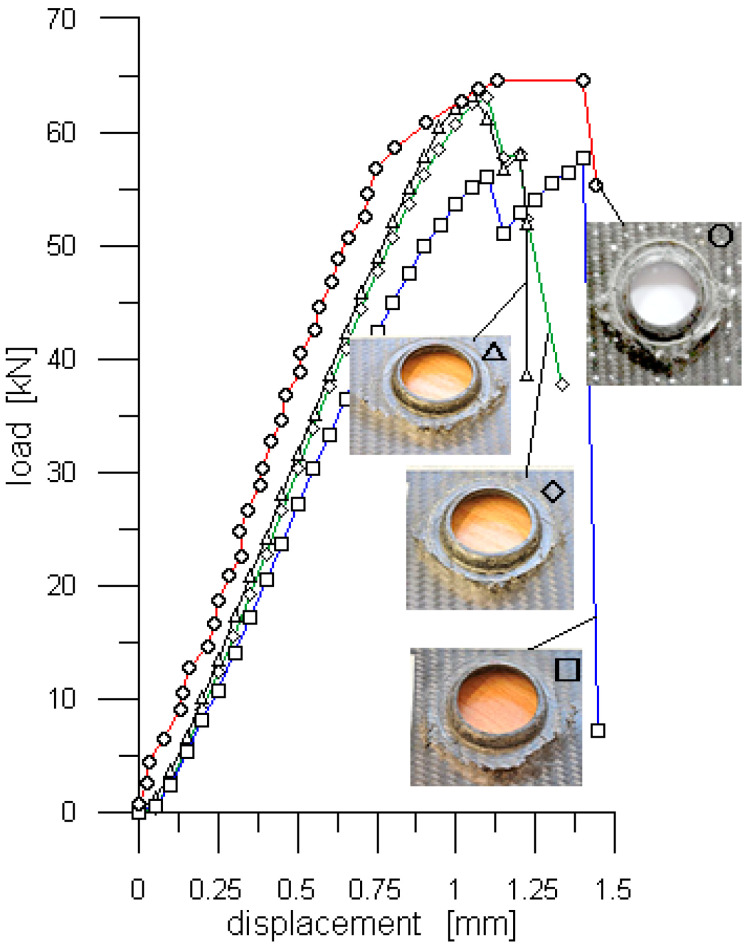
Results of four tests of the ML joint. Symbol “O” indicates the load–displacement curve determined with the help of the DIC and symbols “△”, “◇” and “☐” indicate the load–displacement curves determined with the help of a dial gauge.

To improve the credibility of the findings regarding the load capacity of the ML joint three additional tests of such a joint were run. A dial gauge was used to determine the load–displacement curves. The measurements were taken in the same way as in the case of the SWB joint. The curves based on these measurements are marked with the “△”, “◇” and “☐” symbols, (Figure 10), while the one determined with the help of the DIC with “O” symbol. The corresponding load capacities were 63.3 kN, 63.0 kN, 57.6 kN and 64.5 kN, respectively, and each of them significantly exceeded that of the SWB joint.

**Figure 11:**
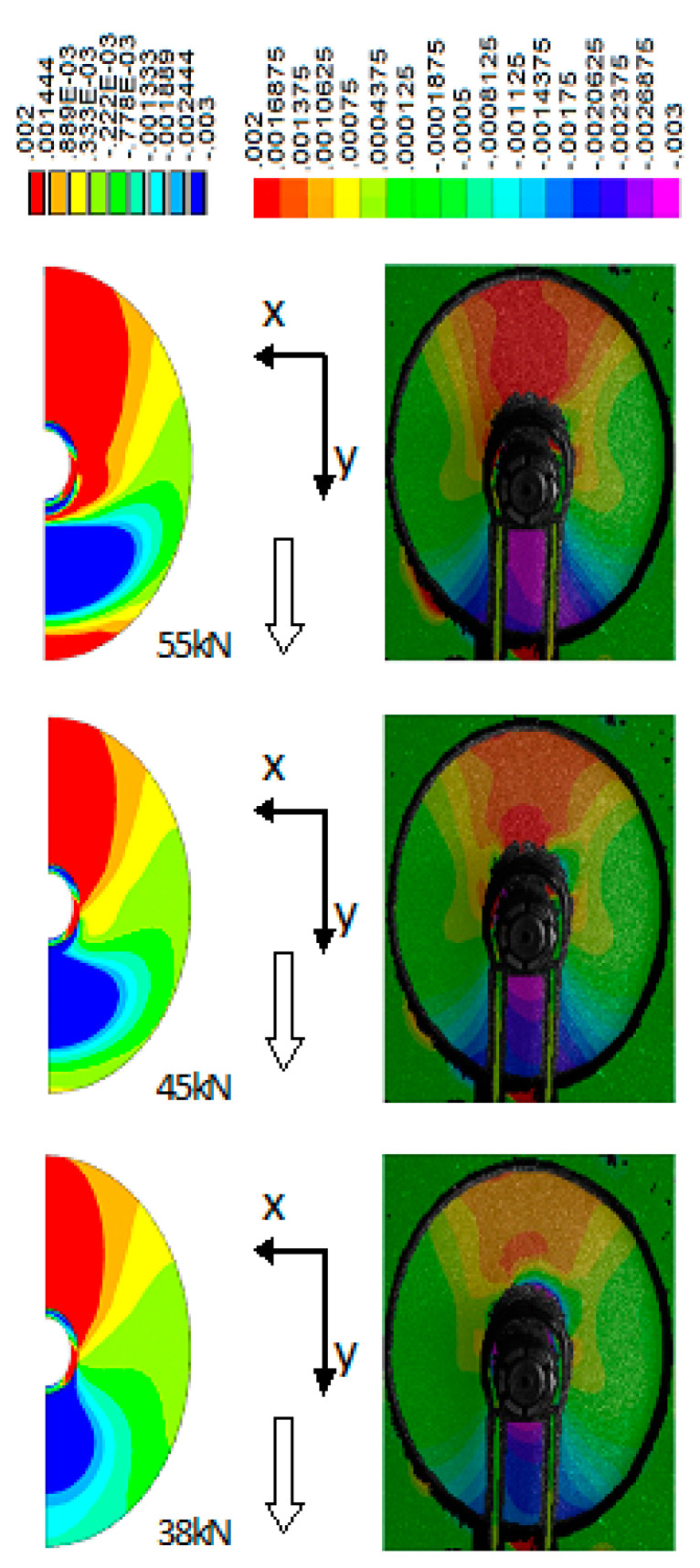
Examples of the ε_yy_ strain fields determined by FE modelling (**left**) and DIC technique (**right**) for 38 kN, 45 kN and 55 kN loadings. The arrows indicate the load direction.

The comparison of the failure loads of the ML and SBW joints indicated that in most cases the load capacity of the former was about 70% higher than that of the latter. This difference can be readily explained after inspection of the ε_yy_ strain fields (Figure 11), determined experimentally for the ML joint with the use of the DIC method and numerically with the previously described FE model. Only the ML joint was modelled, since, in the case of bolt joints, the mechanism of load transfer is well documented in literature, e.g., [30,42] in the case of polymer matrix laminates and, e.g., refs. [33,43] in the case of hybrid laminates. Downward loading of the ML joint produced compressive strain in the front of the joint (the zone of the plate below the joint) and tensile strain behind it (the zone of the plate above the joint), as indicated by both the experimental and numerical analysis (Figure 11). Such strain pattern indicated that the load was transferred into the plate with the front and rear parts of the ML joint as opposed to a typical bolt joint which can transfer a load with its front part only, no matter whether the load is transferred into a polymer matrix laminate [30,42] or a hybrid one [33,43].

The load transfer mechanism of the ML joint resulted in a more complex failure process than the one typical for bolt joints. In the case of bolt joints, no matter whether a solely polymer matrix laminate or a hybrid one was loaded, there was no evidence of damage present behind the bolt. Instead, extensive bearing damage was present at the front part of the bolt–laminate interface. In some cases, it was accompanied by a tensile fracture of a varying extent, originating at the side edges of the hole and propagating in an approximately transverse direction relative to the loading direction [30,31,42,43,44]. With regard to the proportion of the tensile and bearing failure, the failure pattern changed depending on the lay-up and the distance between the bolt and plate edges. 

**Figure 12:**
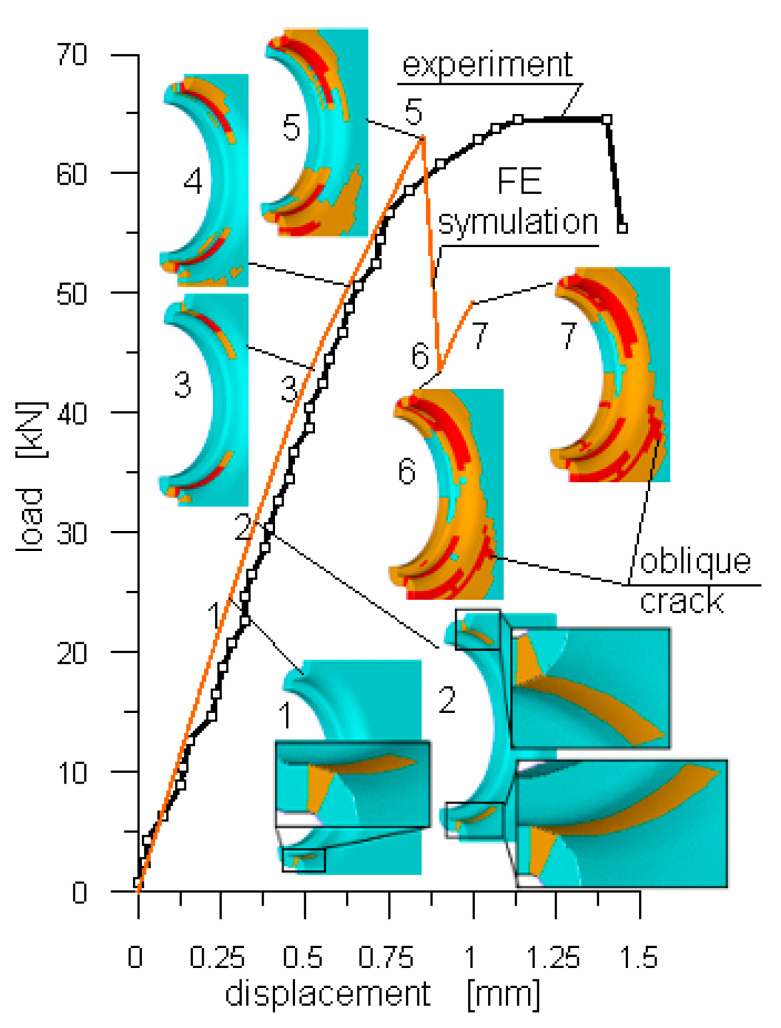
Experimental (determined with DIC) and calculated (FE) load–displacement curves. Pictures 1–7 present increasing extent of damage. Cyan colour indicates intact elements, orange—the elements with several damaged layers, while red indicates the elements in with all layers were damaged.

In the case of the ML joint, as indicated by the results of numerical modelling, (Figure 12), the damage started in the front of the joint, (picture no.1), but after a small increase in the displacement, the damage started to develop at the back of the joint as well, (picture no.2). A further increase in the displacement resulted in the damage growth along the joint perimeter and outwards (pictures no.3 and 4). At the failure load, (picture no.5), a slight increase in the damage of the laminate was observed at the joint’s back. A further increase in the displacement resulted in the significant load drop followed by the initiation of an oblique crack, (picture no.6), and the slight increase in force, and further development of the oblique crack, (picture no.7). Unfortunately, a further increase in the displacement resulted in lack of convergence and the damage simulation was stopped. 

**Figure 13:**
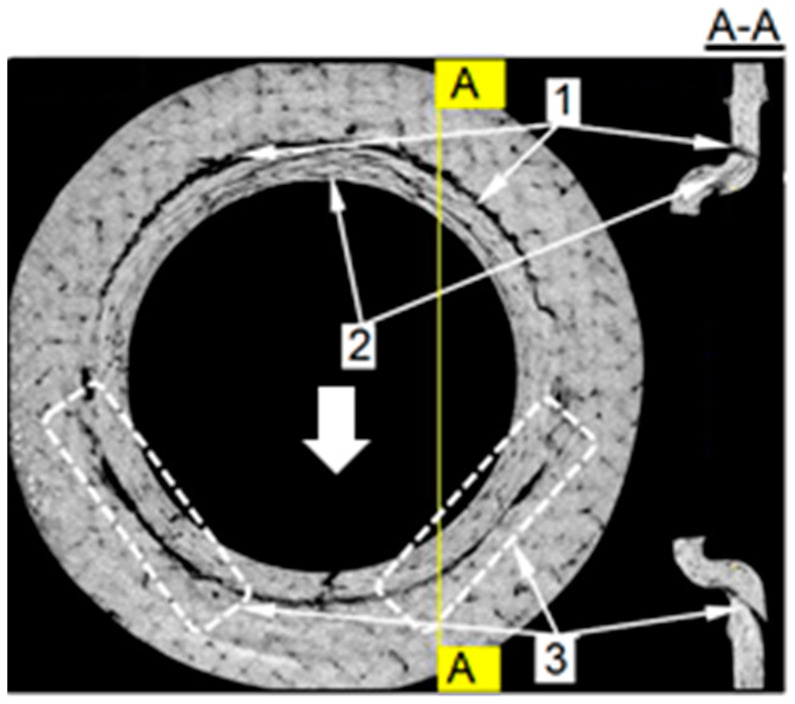
Damage of the laminate produced by the ML joint. The arrow indicates the loading direction.

The additional information about the damage of the laminate surrounding the ML joint was deduced from the X-ray picture obtained with the CT. Two joint cross-sections are presented, Figure 13: in the plane of the plate (on the left-hand side) and in the plane parallel to the load direction, and perpendicular to the plane of the plate, denoted A–A. The left one was located approximately in the middle of the plate thickness, and exposed cracks in the front of the joint (3) and behind it (1). Additional information was provided by the A–A cross-section. It showed that the surface of the rear crack (1) was approximately perpendicular to the midplane of the plate, suggesting tensile failure. The surface of the front crack was oblique. Such a macroscale configuration of the crack surface suggested compressive failure, which could be precluded by the local micro delamination, micro buckling of reinforcement and kink bands formation. For the investigated problem, the micro mechanism of a compressive crack formation itself was beyond the scope of interest. Readers interested in micro mechanism of compressive failure can find detailed information concerning this issue in, e.g., [45,46,47]. 

**Figure 14:**
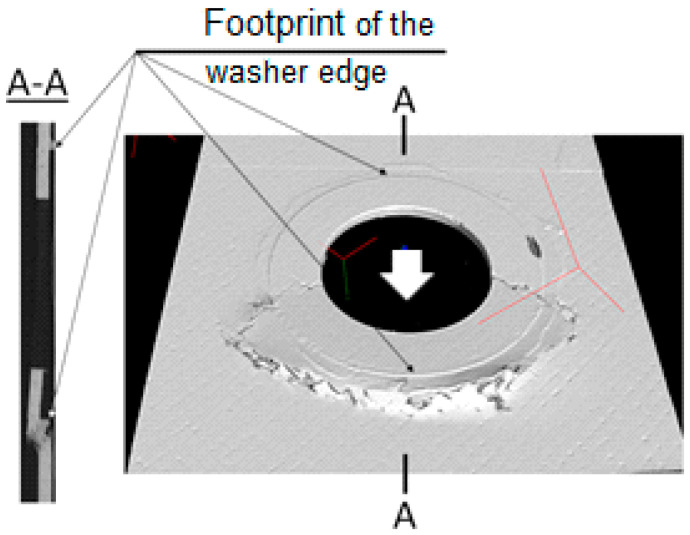
Damage of the laminate produced by the SBW joint. The arrow indicates the loading direction.

The CT X-ray picture of the damage produced to the plate by the SBW joint is presented in Figure 14. It can readily be observed that no evidence of failure produced by the SBW joint was found behind the bolt.

## 4. Conclusions

A particular mechanism of load transfer, load capacity, damage and associated failure modes of the original ML joint, intended for in-plane loading of thin laminate plates with concentrated loads, were investigated experimentally and numerically. 

It was shown that:

- the ML joint offered about 70% higher load capacity compared to that of a SBW joint of the same relevant geometrical parameters.
- such a relatively high-load capacity was due to the load transfer mechanism specific to the ML joint. This mechanism meant that the ML joint could transfer a load with its front and rear parts as opposed to a bolt joint which transferred a load with the contact pressure developed at the front part of the laminate—bolt interface only. Therefore, in the case of laminates with polymer matrixes, the former could serve its purpose much better than the latter.
- the final damage extent determined with, (i) the use of the original algorithm incorporated in the commercial ANSYS FE code and, (ii) the use of CT (Figure 13) was very alike, (picture no.7 in Figure 12). This evidenced, to a large extent, the credibility of the developed algorithm and its usefulness.

The understanding of the load transfer mechanism provided some background for future investigation aiming for optimization of the geometry of metal parts.

## Figures and Tables

**Table 1:** Mechanical properties of a single laminate layer (Advanced Composite Group MTM46 Data Sheet).

Elastic Constants MPa	Strength MPa
E_11_ = E_22_ = 55,800 *	X_T_ = 497
	Xc = 698
E_3_ = 9200 **	Y_T_ = 513
G_12_ = 3510	Y_C_ = 706
G_23_ = 2210 **	Zt = 59.8 **
G_31_ = 2750 **	Zc = 813 **
ν_12_ = 0.04	S_12_ = 113
ν_23_ = ν_13_ = 0.3 **	S_13_ = S_23_ = 104 **

* average of E_weft_ and E_warp_; ** estimated based on [40,41].

## Data Availability

The data presented in this study are available on request from the corresponding author.
